# The Use of Mosquito Nets and the Prevalence of *Plasmodium falciparum* Infection in Rural South Central Somalia

**DOI:** 10.1371/journal.pone.0002081

**Published:** 2008-05-07

**Authors:** Abdisalan M. Noor, Grainne Moloney, Mohamed Borle, Greg W. Fegan, Tanya Shewchuk, Robert W. Snow

**Affiliations:** 1 Malaria Public Health & Epidemiology Group, Centre for Geographic Medicine Research-Coast, Kenya Medical Research Institute/Wellcome Trust Research Programme, Nairobi, Kenya; 2 Centre for Tropical Medicine, University of Oxford, John Radcliffe Hospital, Headington, Oxford, United Kingdom; 3 Food Security Analysis Unit- Somalia, Parklands, Nairobi, Kenya; 4 Infectious Diseases Epidemiology Unit, Department of Epidemiology and Population Health, London School of Hygiene and Tropical Medicine, London, United Kingdom; 5 United Nations Children's Fund, Somalia Support Centre, Nairobi, Kenya; Canadian Agency for Drugs and Technologies in Health, Canada

## Abstract

**Background:**

There have been resurgent efforts in Africa to estimate the public health impact of malaria control interventions such as insecticide treated nets (ITNs) following substantial investments in scaling-up coverage in the last five years. Little is known, however, on the effectiveness of ITN in areas of Africa that support low transmission. This hinders the accurate estimation of impact of ITN use on disease burden and its cost-effectiveness in low transmission settings.

**Methods and Principal Findings:**

Using a stratified two-stage cluster sample design, four cross-sectional studies were undertaken between March-June 2007 across three livelihood groups in an area of low intensity malaria transmission in South Central Somalia. Information on bed net use; age; and sex of all participants were recorded. A finger prick blood sample was taken from participants to examine for parasitaemia. Mantel-Haenzel methods were used to measure the effect of net use on parasitaemia adjusting for livelihood; age; and sex. A total of 10,587 individuals of all ages were seen of which 10,359 provided full information. Overall net use and parasite prevalence were 12.4% and 15.7% respectively. Age-specific protective effectiveness (PE) of bed net ranged from 39% among <5 years to 72% among 5–14 years old. Overall PE of bed nets was 54% (95% confidence interval 44%–63%) after adjusting for livelihood; sex; and age.

**Conclusions and Significance:**

Bed nets confer high protection against parasite infection in South Central Somalia. In such areas where baseline transmission is low, however, the absolute reductions in parasitaemia due to wide-scale net use will be relatively small raising questions on the cost-effectiveness of covering millions of people living in such settings in Africa with nets. Further understanding of the progress of disease upon infection against the cost of averting its consequent burden in low transmission areas of Africa is therefore required.

## Introduction

The evidence on the public health impact supporting the wide-scale use of insecticide treated nets (ITNs) in Africa is drawn from areas of stable malaria transmission where *Plasmodium falciparum* infection prevalence in the community is often over 40% [1], [2]. There is a paucity of parasitological or health impact data on the benefits of net/ITN in areas of Africa that support low stable or unstable transmission.

Across the horn of Africa the dominant vector is *Anopheles arabiensis* [3], a less efficient vector compared to its sibling species across the central belt of Africa, *An. gambiae ss*. The semi-arid conditions of large parts of northern Sudan, Ethiopia, Eritrea, Djibouti and Somalia support malaria transmission conditions that result in low *P. falciparum* parasite prevalence among resident communities [4]. Surprisingly little is known about the malaria infection and disease epidemiology in the semi-arid settings of the East and Horn of Africa, with the exception of studies in Eastern Sudan [5]–[8]. Areas of low, stable or unstable malaria transmission provide different challenges for prevention and control strategies to those prescribed for more stable transmission areas. Where parasite exposure is infrequent the clinical consequences of *P. falciparum* infection are more likely to directly relate to the risk of infection compared to areas of high intensity transmission where the cumulative effects of repeated infection on the development of clinical immunity are more pronounced [9]. The impact and recommendations for the deployment of ITN in these areas of Africa remains unclear.

With the fall of the government in 1989, Somalia has been without a central authority and has suffered the ravages of civil war. In this fragile setting several international relief agencies and non-governmental organizations currently support the national ministries of health of the three self-declared states of South-Central, Puntland and Somaliland in the delivery of preventative and curative services [10]. In 2004, the Global Fund for Aids, TB and Malaria (GFATM), awarded Somalia USD 12.8 million to support the new national malaria control strategy [11], [12] With these funds the United Nations Children's Fund (UNICEF) coordinated various partners and the ministries of health to provide over 700,000 free and/or highly subsidised ITNs by mid 2007 [13], [14]. Here we report the parasitological impact of net use in 2007 from a series of community-based surveys undertaken in an area of low intensity malaria transmission in South Central Somalia.

## Method

### Objectives

The objective of this study was to examine the effectiveness of mosquito bednets delivered under routine operational conditions in South-Central Somalia, an area of generally low malaria transmission.

### Participants

**Figure 1:**
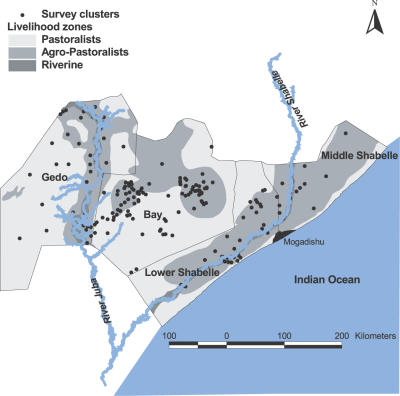
Map of South-Central Somalia regions showing the distribution of clusters where surveys were undertaken by livelihood zone. 6/197 clusters could not be geo-located and therefore not shown in the figure.

The Food and Agriculture Organization-Food Security Analysis Unit (FAO-FSAU) has undertaken regular surveys since 1995 in all regions of Somalia to monitor the nutritional status of children less than 5 years of age and internally displaced groups [15]. In 2007, four cross-sectional nutritional survey rounds were undertaken by FAO-FSAU in the regions of Bay (March, April-May) and Gedo (June), Middle Shabelle and Lower Shabelle (June) of South Central Somalia (Figure 1). UNICEF and the World Health Organization (WHO) requested that in addition to routine nutrition data collection FAO-FSAU undertook investigations of malaria prevalence and bed net use among individuals of all age groups.

The four survey regions (Figure 1) are densely populated with an estimated 1.6 million people located between the two major rivers (the Shabelle and Juba) that transect the southern part of Somalia. The only published malaria surveys reported from this area derive from work by Warsame and colleagues in the late 1980's where *P. falciparum* infection prevalence among children aged 1–9 years was 18% in Lower Shabelle [16]. *Anopheles arabiensis* is the only reported malaria vector in the area [17]. A UNICEF multiple indicators cluster survey (MICs) showed that bed net coverage among children aged less than 5 years in August 2006 in the four study regions was 8.3% with 84.7% of all nets being ITN of which 88.1% were long-lasting insecticidal nets [18]. Southern Somalia has a savannah climate with the long rains beginning in March-April. The flat irrigated land between the two perennial rivers, however, provides fertile land for crops (maize and sorghum). Floods in this area are a relatively common occurrence.

### Description of procedures or Investigations undertaken

A stratified two-stage cluster sampling design was used whereby for each survey sampling was stratified by livelihood zone: pastoral; agro-pastoral; and riverine. A livelihood zone refers to a specific food economy group in which communities share similar types and levels of assets; similar options for production and income generation; and often with similar socio-cultural and environment profile and are therefore vulnerable to similar risks [19]. Communities were defined as pastoral if they engaged primarily in livestock production and were nomadic (moved with their livestock from place to place in search of water and pasture); agro-pastoral if they practiced mixed crop and livestock production; and riverine if they lived along the river and were primarily involved in crop production and river-based economy. Lower and Middle Shabelle regions were combined and samples derived for agro-pastoralist and riverine zones; Bay had pastoralist and agro-pastoralist zones; and Gedo had all three zones resulting in a total of seven livelihood zones. In each livelihood zone, 30 rural clusters were first randomly sampled and within each cluster, 30 households were further randomly sampled. The 210 sampled clusters were derived from a sampling frame of settlements and populations for each livelihood developed by the United Nations Development (UNDP) project and UNICEF/WHO for polio vaccination campaigns collàted by the FAO Somali Water and Land Information Management (SWALIM) and supplemented with additional information from field visits [20]. Survey tools, which were based on the standard MICs bednet and parasitological questions [18], were pre-tested for internal consistency and validity through dummy interviews and pilot field surveys. During field work each household head was approached by an interviewer, trained in survey procedures and extraction of blood samples, who explained the purpose of the surveys and obtained a household verbal consent.

Following the completion of the nutritional assessment in each cluster, community members were further investigated as part of the malaria assessment survey. Sampling continued from one randomly selected household to the next across the cluster until approximately fifty people had been examined. Respondents were asked about the use of a mosquito net during the night prior to the survey day and provided a finger prick blood sample that was examined for the presence of *P. falciparum* infection using a rapid diagnostic test (RDT) (Paracheck Pf ™, Orchid Biomedical Systems, Goa, India). Unfortunately no information was collected on whether the net had been treated with an insecticide nor where the nets were obtained from, we revisit this is the discussion and interpretation of the results. Additional information was recorded on the age and sex of survey participants. All Individuals found to have a positive RDT result were treated with nationally recommended age-weight specific sulphadoxine-pyrimethamine artesunate combination therapy.

### Ethical Approval

Ethical approval was provided through permission by the Ministry of Health Somalia, Transitional Federal Government of Somalia Republic, Ref: MOH/WC/XA/146./07, dated 02/02/07. Informed verbal consent was sought from all participating households and individuals.

### Statistical methods

Data entry and storage was undertaken using EPI Info (Epi Info™ Version 6, CDC, USA), through customized data entry screens with in-built range and consistency checks. Each household member was assigned a unique identifier. Descriptive summaries of infection prevalence by bed net use and community-level covariates were generated using STATA version 9.2 (Statacorp 2003, College Station, USA) and MS Excel 2007 (Microsoft, Redmond, USA). To account for the clustered nature of the data, the *svy* command in STATA was used with the cluster as the primary sampling unit *(psu)* stratified by livelihood zones. All results were weighted (weight = 1/probability of selection) to account for unequal probabilities of selection of clusters across livelihood zones. To test for differences in proportions in net use or infection prevalence a Pearson χ^2^ test accounting for survey design (clustering and stratification) was used and the test statistic converted to an F-statistic using the second-order Rao and Scott (1984) correction yielding wider confidence intervals and conservative P-values compared to the uncorrected χ^2^ test.

For each livelihood data the *mhodds* command in STATA 9.2 was used to examine the association between parasite infection and sleeping under a bed net the night before survey, adjusting for the separate confounding effects of livelihoods, age and sex using the Mantel-Haenszel method. Odds ratios; their 95% confidence intervals (CI); and associated P-values for the Mantel-Haenszel χ^2^ test are reported.

## Results

Of the sampled 210 clusters, 201 were surveyed and 9 pastoralist clusters could not be located during survey. A total 10,587 individuals were seen during the four surveys in 201 clusters (Figure 1). A total of 132 (1.2%) RDT results were invalid and recorded as un-interpretable. A further 96 (0.9%) respondents had no information recorded on net use the previous night. These 228 (2.1%) individuals from 4 clusters have been excluded from the subsequent analysis. The distribution of the remaining 10,359 respondents from 197 clusters who provided a valid RDT and net use result by livelihood, age and sex are shown in Table 1.

**Table 1:** Characteristics of 10,359 survey participants who provided a valid RDT result and whose bed net use was known in 197 clusters of South Central Somalia

	% (n)	Sleeping under a bed net, % (95% CI) [n]	Total *Pf* positive % (95% CI) [n]	*Pf* positive sleeping under a bed net, % (95% CI) [n]	*Pf* positive not sleeping under a bed net, % (95% CI) [n]
**Overall**	10,359	12.4 (8.4–16.5) [1,418]	15.7 (12.8–18.6) [1,603]	6.9 (2.7–11.1) [115]	17.0 (13.9–20.0) [1,488]
**Type of dominant type livelihood**					
Pastoralists	30.1 (3,113)	8.6 (4.5–12.6) [319]	24.1 (17.8–30.4) [667]	11.6 (5.3–17.9) [34]	25.3 (18.7–31.8) [633]
Agro-pastoralist	49.9 (5,165)	12.6 (6.4–18.8) [679]	13.4 (10.1–16.7) [698]	6.3 (0.0–12.6) [52]	14.4 (11.0–17.9) [646]
Riverine	20.1 (2,081)	20.9 (13.1–28.7) [420]	7.2 (3.2–11.2) [238]	4.2 (0.1–7.6) [29]	7.9 (3.4–12.5) [209]
**Age category (years)** *					
<5	30.9 (3,202)	12.4 (8.4–16.3) [444]	19.6 (15.6–23.5) [598]	10.0 (2.8–17.3) [51]	20.9 (16.8–25.0) [547]
5–14	27.0 (2,799)	14.0 (8.7–19.2) [433]	20.5 (16.7–24.3) [547]	6.2 (1.8–10.7) [31]	22.8 (18.8–26.8) [516]
>14	42.1 (4,358)	11.5 (7.8–15.3) [541]	9.8 (7.4–12.2) [458]	5.0 (2.3–7.6) [33]	10.5 (7.9–13.0) [425]
**Gender**					
Male	48.2 (4,997)	12.3 (8.1–16.5) [679]	17.0 (13.9–20.0) [832]	7.7 (2.6–12.8) [60]	18.3(15.0–21.5) [772]
Female	51.8 (5,362)	12.6 (8.6–16.5) [739]	14.5 (11.6–17.5) [771]	6.1 (2.4–9.8) [55]	15.7 (12.6–18.9) [716]

* age of 47 individuals was missing

* Proportions and their precisions have been adjusted for clustering and stratification and are weighted using the inverse probability of selection of a cluster within a livelihood.

The overall reported use of nets during the night prior to the survey was 12.4% (95% CI: 8.4–16.5) (Table 1). Net use was significantly higher among communities classified as riverine (20.9%, 13.1–28.7) compared to pastoralists (8.6%; F_1, 96_ = 9.0, P = 0.004) or agro-pastoralists although the difference was not significant for the latter (12.6%; F_1, 134_ = 2.7, P = 0.104). Children under the age of five years had similar net use compared to children 5–14 years of age (F_1, 193_ = 1.9, P = 0.174) and adults (F_1, 194_ = 0.6, P = 0.438) (Table 1). However evidence of a difference in net use by children 5–14 years of age was very weak compared to adults (Table 1: F_1, 194_ = 3.5, P = 0.061). There was no significant difference in reported net use by sex (Table 1: F_1, 194_ = 0.1, P = 0.705).

Overall *P. falciparum* infection prevalence was 15.7% (95% CI: 12.8–18.6) (Table 1). The highest prevalence was recorded among the pastoralist communities (24.1%) and the lowest prevalence among riverine communities (7.2%; F_1, 96_ = 18.3, P<0.001). Infection prevalence was significantly lower among adults (9.8%) compared to children under five years of age (19.6%; F_1, 194_ = 56.0, P<0.001) and children 5–14 years (20.5%; F_1, 194_ = 55.7, P<0.001). There was no significant difference in infection prevalence among children aged below five years compared to those 5–14 years (F_1, 193_ = 0.4, P = 0.518). Infection prevalence was higher among males (17.0%) compared to females (14.5%; F_1, 194_ = 9.3, P = 0.003).

Table 1 also summarizes the prevalence of infection by use of bed nets. Overall infection prevalence was significantly lower among net users: 6.9% (95% CI: 2.7–11.1) compared to non-net users 17.0% (95% CI: 13.9–20.0); (F_1, 194 = _9.9, P<0.002). Across all livelihoods, age and sex categories prevalence was significantly lower among net users although among the riverine communities the evidence for this was very weak (F_1, 36 = _3.7, P = 0.062).

To examine in more detail the effects of net use on *P. falciparum* infection prevalence Mantel-Haenszel odds ratios were calculated that adjust for the effects of age and sex within each livelihood grouping (Table 2). Within each livelihood group the use of nets significantly reduced the chances of being infected with *P. falciparum* with an adjusted protective effectiveness of between 60% (95% CI: 42–72, P<0.001) among pastoralist communities to 48% (95% CI: 22–65, P = 0.001) among riverine communities (Table 2). When all data were combined and adjusted for livelihood and sex, bed nets reduced significantly the probability of infection in each age group, with a protective effectiveness among children 5–14 years of age of 71% (95% CI: 57–80, P<0.001); followed by adults (48%, 95% CI: 24–64, P<0.001); and children under five years of age (39%, 95% CI: 17–55, P = 0.002). Overall, after adjusting for livelihood, sex and age the use of bed nets had a protective effectiveness against parasite infection of (54%, 95% CI: 44–63, P<0.001).

**Table 2:** Results of the Mantel-Haenszel adjusted odds ratios of using bed nets on *Pf* infection by livelihood adjusting for the confounding effect of age and sex.

Livelihood	Age in years	% sleeping under a bed net the night before survey who were positive for parasite infection (n/N)	% NOT sleeping under a bed net the night before survey who were positive for parasite infection (n/N)	Adjusted* Odds Ratio, (95% CI), χ^2^ (df), P-value	Overall adjusted** Odds Ratio, (95% CI), χ^2^ (df), P-value
**Pastoralists**	**<5**	19.2 (15/78)	29.0 (228/787)	**0.58** (0.33–1.04) 3.4 (1), P = 0.068	0.40 (0.28–0.58) 25.0 (5), P<0.001
	**5–14**	9.0 (9/100)	29.3 (227/774)	**0.24** (0.12–0.49) 18.4 (1), P<0.001	
	**>14**	7.1 (10/141)	14.4 (178/1,233)	**0.45** (0.23–0.88) 5.8 (1), P = 0.06	
**Agro-pastoralists**	**<5**	12.2 (24/197)	18.9 (257/1363)	**0.60** (0.38 –0.94) 5.2 (1), P = 0.023	0.47 (0.35–0.63) 26.4 (5), (P<0.001)
	**5–14**	6.2 (14/225)	19.0 (221/1,162)	**0.28** (0.16–0.48) 21.9 (1), P<0.001	
	**>14**	5.4 (14/257)	8.6 (168/1,961)	**0.61** (0.36–1.10) 2.7(1), P = 0.103	
**Riverine**	**<5**	7.1 (12/169)	10.2 (62/608)	**0.68** (0.36–1.28) 1.5 (1), (P = 0.223)	0.52 (0.35–0.78) 10.5 (5), (P = 0.001)
	**5–14**	7.4 (8/108)	15.8 (68/430)	**0.42** (0.20–0.92) 5.1 (1), P = 0.024	
	**>14**	6.3 (9/143)	12.7 (79/623)	**0.48** (0.23–0.97) 4.3 (1), P = 0.037	
**Combined data**	**<5**	11.5 (51/444)	19.8 (547/2,758)	**0.61** (0.45–0.83) 10.0 (1), P<0.001	0.46 (0.37–0.56) 61.2 (17), P<0.001
	**5–14**	7.2 (31/433)	21.8 (516/2,366)	**0.29** (0.20–0.43) 44.6 (1), P<0.001	
	**>14**	6.1 (33/541)	11.1 (425/3,817)	**0.52** (0.36–0.75) 12.3 (1), P<0.001	

* The age-specific Odds Ratios within each livelihood were adjusted for sex

** Within each livelihood the overall Odds were adjusted for both age and sex. For the combined data the odds were adjusted for livelihood, age and sex

## Discussion

There are remarkably few studies on the impact of ITN on infection prevalence among African communities living under conditions of low transmission [21] and none we can identify undertaken in the semi-arid areas of Africa where transmission is maintained by *An. arabiensis*. After a limited period of national net distribution in Somalia we have shown that among the varied communities of South Central Somalia net use remains low with only 12.4% of residents reporting using a net (Table 1). Among children aged below 5 years 12.4% (Table 1) were reported using a net compared to only 8.7% reported during a cluster sample survey in the same regions 8–10 months earlier [18]. Among non-net users *P. falciparum* infection prevalence was overall 17.0% ranging from 7.9% among the riverine communities to 25.3% among the pastoralists. We cannot explain the paradoxical observation that communities closer to rivers had lower infection prevalence and would require a more comprehensive vector biting and larval entomological survey to explore this further. Similar findings, however, have been reported in The Gambia where communities living near mosquito breeding sites had proportionately lower parasite rates [22], [23]. Nevertheless the ranges of reported prevalence support the view that transmission intensity in this area of Somalia is between hypo- and mesoendemic. The overall age, sex and livelihood adjusted protective effect of nets among these communities was 54% ranging from 60% among pastoralist communities to 48% among communities located along the rivers (Table 2). The largest effects were seen among pastoralist and agro-pastoralist children aged 5–14 years. We suspect that most of these nets were treated with insecticide and the majority of those treated were LLIN (see methods), however, the specifics of net treatment were not recorded.

The protective effect of nets on infection prevalence among the sampled populations in South Central Somalia was consistent across age-groups (Table 2). Given the relatively higher prevalence of infection through older childhood and into adulthood it is important to recognize the need to provide ITN to all members of a community and not focus only on young children in areas of low transmission. This resonates with recent calls for high coverage among all community members across the range of transmission settings [24] where it is also recognized that individuals older that five years contribute to transmission.

Several studies in stable, hyper- holoendemic areas of Africa have examined the impact of nets and ITN on parasite prevalence. The meta-analysis of randomized controlled trials (RCT) suggests that the median protective effect of ITN on infection prevalence in children aged less than 15 years is only 13% [1]. Including an examination of non-RCT studies it is clear that the ranges of reported protection vary widely largely a result of different studies examining different age groups and using different designs. This makes comparisons difficult and presents several interpretation problems. First, investigations that have been part of randomized controlled trials, where efforts are made to maximize coverage of net or ITN use among communities neighboring “control” communities may have under-estimated the effects upon infection prevalence [25]–[30] due to a shadowed, wide-area protection provided by the intervention areas across control areas [25]–[32]. Second, the combined active clinical detection surveillance and treatment among the same children examined for the prevalence of infection will bias the results away from significance as more control children will receive effective treatment during the surveillance period if nets protect against clinical events [27], [28], [30], [33]–[35]. These combined effects might explain why several investigations using single cross-sectional studies of infection prevalence against the reported use of nets and ITN under routine operational delivery conditions have tended to provide higher estimates of protection (51–63%) than those described during an RCT design [21], [36]. Finally, studies that have examined reductions in parasite prevalence due to nets or ITN among older children in high transmission areas will have been unable to identify the true impact on the incidence of infection through a simple cross-sectional estimate of prevalence [27], [28], [33], [35]. The duration of sub-patent or un-treated infections in an individual host may be many months making the distinction between old and new infections difficult due to saturation of multiple infections in high transmission areas. Conversely, in very young, immunologically semi-naive infants at Kilifi, on the Kenyan coast, comparisons of those sleeping under ITN with community randomized control infants showed that infection rates were reduced by 50% among those using ITN [37]. Similarly an operational effectiveness study of ITN protection against infection prevalence in children aged less than 24 months in Tanzania showed a 62% protection against infection prevalence [38].

Our findings among a wider age group living in an area with a low rate of parasite exposure might therefore be expected to correspond to levels of protection described among young infants living in much higher transmission settings in Kenya [37] and Tanzania [38]. Our findings are also consistent with observations made during non-RCT investigations of net/ITN use in The Gambia [35] and the unstable, highland areas of Kenya [21]. The review by Lengeler (2004) might have under-estimated the impact on the prevalence of infection consequent upon ITN use. We would argue that the incidence of new infections might be reduced by over 50% in all transmission settings but measurement, through prevalence surveys, is critically dependent upon the selected age range in different areas of differing intensities of malaria transmission.

Halving the risks of *P. falciparum* infection among all age groups through the use of nets in an area of low transmission intensity has not been previously described among the semi-arid areas of Africa. These findings appear at first glance to support the wide-scale use of ITN in these areas. However, coverage remains poor and many more people would need to be reached to achieve a significant population-attributable impact. This then leaves an issue hard to resolve with the current data. Where infection prevalence is very low, such as the communities in the South Central region of Somalia, when does it become cost-ineffective to deliver ITN to the entire population? Covering 1.6 million people with an ITN to reduce infection risks from 17% to 7% might be seen as an expensive way to tackle the problem of malaria in this region. The answers to this dilemma would require a better understanding of the health risks consequent upon infection to compute a cost-per-disability-adjusted-life-year averted and a comparison with other approaches to malaria infection reduction in semi-arid, semi-nomadic and conflict areas of Somalia.

### Limitations

A particular challenge in Somalia, due to the large pastoral community, is maintaining a consistent survey sampling frame and in some cases there are difficulties in locating nomadic pastoralist clusters as seen in this study. To minimize the effect of this, a large sample size was therefore selected. In addition, our estimates of protective effectiveness on infection prevalence might have increased if it were possible to separate out ITN from untreated nets and estimate the impact of ITN alone. We present these estimates of impact under routine delivery conditions where we had no opportunity for strict randomization criteria nor, because of the opportunistic nature of the surveys, have we been able to adjust for the many other potential confounders of infection and net use for example socio-economic status or treatment seeking behaviours. However, the results were internally consistent between groups sharing similar livelihoods and economic activities adding confidence to the observed protective effects.
